# Idiopathic rapid eye movement sleep behaviour disorder: a potential gateway to the development of disease-modifying treatments in neurodegenerative disorders

**DOI:** 10.1007/s00415-016-8235-8

**Published:** 2016-07-14

**Authors:** K. J. Peall, N. R. Robertson

**Affiliations:** Institute of Psychological Medicine and Clinical Neuroscience, University Hospital of Wales, Cardiff University, Heath Park, Cardiff, CF14 4XN UK

## Introduction

Idiopathic rapid eye movement (REM) sleep behavioural disorder (RBD) is classified as one of the REM parasomnias with clinical characteristics including unpleasant dreams, acting out of dreams, and loss of the typical muscle atonia during the REM phase of sleep. Other associated clinical features include olfactory loss, dysautonomia, colour vision impairment and subtle Parkinsonian signs. RBD has been shown to predict development of an alpha-synucleinopathy in 20–45 % of patients within 5 years, and up to 92 % within 14 years post-diagnosis. Hence, this disorder provides a potential opportunity for understanding the prodromal stages of the synucleinopathies and exploring efficacy of potential neuroprotective agents.

## Idiopathic rapid eye movement sleep behaviour disorder: diagnosis, management, and the need for neuroprotective interventions

Iranzo and colleagues provide a concise and readable overview of a clinical area not often familiar to the general neurologist. Their dissection of the disorder into its epidemiology, clinical features and useful research-based questionnaires, alongside the highly relevant and pragmatic diagnostic criteria, differential diagnosis and ‘information to share with the patient’ make this an excellent point to begin reappraising this subject.

Although the exact prevalence of RBD is unknown, consensus opinion suggests that it is under-reported and under-recognised amongst the general population. Questionnaire-based studies in elderly populations (60–97 years) have estimated the rate to be 4.6–7.7 %, whilst polysomnographic studies have produced values of 0.3–1.15 %. Gender appears to be a factor, with >70 % of those diagnosed at specialist sleep units being men, although there is some suggestion that women experience milder symptoms and are, therefore, less likely to seek medical attention.

At its core, RBD involves abnormal sleep behaviours and unpleasant dreams. This review highlights the importance of a collateral history from a bed partner wherever possible. Description of sleep-related activity tends to be one of aggression and fear, beginning abruptly, lasting seconds to minutes, eyes remaining closed and tending to be confined to the bed. If the patient is woken during an event, they tend to be easily orientated and may not recall the nightmare. Injuries to the bed partner and/or patient are not infrequently reported, with protective measures including sleeping in separate rooms to moving furniture away from the bedside. Once started, the clinical course is variable and includes both remission and progression.

Accurate diagnosis of RBD is essential, given the potential neurodegenerative implications discussed below. Polysomnography, ideally with concurrent audio–visual recording, forms the gold standard in diagnostic testing. However, this is rarely offered as a routine form of investigation, is expensive and requires trained specialists. Several questionnaires have been developed, principally the RBD screening questionnaire and RBD single–question screen, both demonstrating high sensitivity but limited specificity at a population level.

It is important to highlight that there are no established guidelines for the treatment of RBD, but that management strategies should focus on reducing the symptoms, discussion of the risk of phenoconversion to one of the synucleinopathies and appropriate clinical follow-up. Symptom management aims to reduce the frequency and intensity of the sleep behaviours either by removal of potentially exacerbating drugs, such as anti-depressants and beta-blockers or instigation of treatment. Anecdotal evidence suggests that clonazepam and melatonin are the most effective, reducing the frequency and intensity of symptoms, although neither of them slows or prevents the onset of further neurodegenerative symptoms.

Sharing of information related to the risk of phenoconversion to a neurodegenerative disorder remains controversial, not least due to the <100 % conversion rate, difficulty in predicting the onset of symptoms and the absence of disease-modifying or curative treatments. However, this review supports the full discussion of potential future symptomatology, highlighting the ease of access to information on the internet and from other sources. The best approach to follow-up of patients with RBD remains uncertain, with access likely to be limited by local availability of resources. Options include discussing potential future symptoms with patients and asking them to contact the department should they develop these, or to provide follow-up appointments to assess for evidence of motor or non-motor symptoms.

Iranzo et al. Lancet Neurol. 2016;15:405–19.

## Visual short-term memory deficits in REM sleep behaviour disorder mirror those in Parkinson’s disease

One of the principal aims of any future neuroprotective treatment would be to delay or prevent the development of cognitive deficits. To allow development testing methods that might enable an early identification of ‘at risk’ individuals, studies have targeted higher risk groups, such as those with *GBA* mutations and idiopathic RBD. Previous cognitive studies have used techniques relying on binary responses, often not allowing the determination of the source of error resulting in a poorer performance. This study used a visual short-term memory (VSTM) task, previously used in *GBA* mutation-positive cohorts, to investigate the precision with which patients with RBD retained items, and whether similar patterns of impairment are seen with *GBA* mutation-negative patients with Parkinson’s disease (PD).

This study included a total of 73 participants: 21 polysomnography-confirmed RBD, 15 drug-naïve PD patients, 11 PD patients receiving oral medical therapy and 26 age and educationally matched healthy controls. All had normal or normal corrected vision, and all patients were *GBA* mutation-negative. All participants were asked to remember a sequence of four coloured bars in different orientation. A single coloured bar would then appear on the screen, and they would be asked to rotate the image to match the original sequence. A series of additional tasks were performed to control for reduced dexterity, temporal decay of information and attention filtering.

Overall, RBD patients demonstrated a similar performance to the *GBA* mutation-negative PD cohort with a significantly lower overall performance (*p* = 0.025 and *p* = 0.003, respectively) and, specifically, a higher proportion of random responses (*p* = 0.001 and *p* = 0.024) compared to controls. The authors suggested that these results potentially reflected that both cohorts shared the same underlying memory impairment and postulated that disruption to cholinergic or dopaminergic neurotransmission was central to this process. However, the most exciting component of this publication is its potential applicability. The authors highlight that the results from the RBD cohort mirror those of *GBA* mutation-negative idiopathic PD patients, unlike the higher rate of misbinding errors seen in *GBA* mutation-positive cohorts. This would imply that patients with RBD form a group which is more representative of the more typical forms of idiopathic PD, and could form the basis for testing of disease-modifying treatments in future clinical trials.

Rolinski et al. Brain 2016;139:47–53.

## The coeruleus/subcoeruleus complex in idiopathic rapid eye movement sleep behaviour disorder

Published in the same edition as the previous article, Ehrminger and colleagues set out to identify the central neuroanatomical regions in RBD pathogenesis, as well as a potential diagnostic test with utility on an individual patient basis. Collectively, the locus coeruleus and subcoeruleus form an adjacent complex, thought to be involved early in the Braak staging of alpha-synuclein disease in human brains. This group had already shown a reduction in neuromelanin signal, proportional to the loss of muscle atonia during REM sleep using a similar imaging modality when comparing PD patients with RBD to healthy controls.

Twenty-one patients with RBD were compared to an equal number of age- and gender-matched controls. Each underwent an assessment of motor, cognitive, sensory and autonomic systems, and questionnaires were used to assess RBD-type symptoms. Cerebral imaging involved 3 T MRI head T1-weighted neuromelanin-sensitive images, analysed by dividing the coeruleus/subcoeruleus complex into three regions, the most rostral acting as a reference point and the other two corresponding to the bilateral regions. Two neuroradiologists, blinded to the clinical findings of the participant, compared the intensity of ten connected voxels from each of the bilateral regions and compared them to the reference region. They were asked to rate each as demonstrating: bilateral complex signal present, signal possibly present or signal low/absent.

Overall, neuromelanin-sensitive intensities were decreased in patients compared to controls, with individual signal reductions demonstrating an 82.5 % sensitivity, 81 % specificity and 80.4 % positive predictive value. Signal intensity was negatively correlated with the percentage of REM sleep without atonia and positively correlated with RBD symptom severity and sleep quality score. The degree of signal loss in this cohort was comparable to that seen in PD patients with RBD symptoms, with similar correlations between signal loss and loss of muscle atonia during REM sleep in both groups (*r* = −0.44 RBD, *r* = −0.49 PD with RBD cohort). The authors suggested that these findings supported a progressive dysfunction of the system involved in REM atonia and directly linked to the clinical severity of RBD.

Results from this study, other in vivo imaging work demonstrating changes to diffusion metrics in the midbrain and rostral pons and post-mortem evidence of early involvement of this region with alpha-synuclein deposition suggest that this region is pivotal in the development of RBD symptomatology. The authors acknowledge that this imaging focuses on the combined coeruleus/subcoeruleus complex, with the noradrenergic arousal system contained in the former. This technique does not facilitate the dissection of exact anatomical or neuron-type involved in the process, and it remains a possibility that there is co-degeneration of the noradrenergic neurons. Clearly, further longitudinal studies need to be undertaken to fully understand any potential progressive change in the imaging characteristics of this region. However, the potential of a both sensitive and specific, individual patient, non-invasive testing method that could be used early in the disease process, provides huge potential not only in neuroprotective intervention but also as an outcome marker in monitoring progress in future clinical trials.

Ehrminger et al. Brain 2016;139:1180–88.

